# A dosimetric evaluation of knowledge‐based VMAT planning with simultaneous integrated boosting for rectal cancer patients

**DOI:** 10.1120/jacmp.v17i6.6410

**Published:** 2016-11-08

**Authors:** Hao Wu, Fan Jiang, Haizhen Yue, Sha Li, Yibao Zhang

**Affiliations:** ^1^ Key Laboratory of Carcinogenesis and Translational Research (Ministry of Education/Beijing) Department of Radiation Oncology, Peking University Cancer Hospital & Institute Beijing China

**Keywords:** knowledge‐based planning, RapidPlan, rectal cancer, VMAT, SIB

## Abstract

RapidPlan, a commercial knowledge‐based optimizer, has been tested on head and neck, lung, esophageal, breast, liver, and prostate cancer patients. To appraise its performance on VMAT planning with simultaneous integrated boosting (SIB) for rectal cancer, this study configured a DVH (dose‐volume histogram) estimation model consisting 80 best‐effort manual cases of this type. Using the model‐ generated objectives, the MLC (multileaf collimator) sequences of other 70 clinically approved plans were reoptimized, while the remaining parameters, such as field geometry and photon energy, were maintained. Dosimetric outcomes were assessed by comparing homogeneity index (HI), conformal index (CI), hot spots (volumes receiving over 107% of the prescribed dose, $V_{107\%}$), mean dose and dose to the 50% volume of femoral head ($D_{\text{mean}\_\text{FH}}$ and $D_{50\%\_\text{FH}}$), and urinary bladder ($D_{\text{mean}\_\text{UB}}$ and $D_{50\%\_\text{UB}}$), and the mean DVH plotting. Paired samples *t*‐test or Wilcoxon signed‐rank test suggested that comparable CI were achieved by RapidPlan (0.99 ± 0.04 for ${\text{PTV}}_{\text{boost}}$, and 1.03 ± 0.02 for PTV) and original plans (1.00 ± 0.05 for ${\text{PTV}}_{\text{boost}}$ and 1.03 ± 0.02 for PTV), respectively (p > 0.05). Slightly improved HI of planning target volume $\left({\text{PTV}}_{\text{boost}}\right)$ and PTV were observed in the RapidPlan cases (0.05 ± 0.01 for ${\text{PTV}}_{\text{boost}}$, and 0.26 ± 0.01 for PTV) than the original plans (0.06 ± 0.01 for ${\text{PTV}}_{\text{boost}}$ and 0.26 ± 0.01 for PTV), p < 0.05. More cases with positive $V_{107\%}$ were found in the original (18 plans) than the RapidPlan group (none). RapidPlan significantly reduced the $D_{50\%\_\text{FH}}$ (by 1.53 Gy/9.86% from 15.52 ± 2.17 to 13.99 ± 1.16 Gy), $D_{\text{mean}\_\text{FH}}$ (by 1.29 Gy/7.78% from 16.59±2.07 to 15.30±0.70 G), $D_{50\%\_\text{UB}}$ (by 4.93 Gy/17.50% from 28.17±3.07 to 23.24±2.13 Gy), and $D_{\text{mean}\_\text{UB}}$ (by 3.94 Gy/13.43% from 29.34±2.34 to 25.40±1.36 Gy), respectively. The more concentrated distribution of RapidPlan data points indicated an enhanced consistency of plan quality.

PACS number(s): 87.55.de; 87.55.dk

## I. INTRODUCTION

As reported by many inhouse approaches, knowledge‐based radiotherapy (KBRT) treatment planning is deemed to reduce the interplanner varieties of plan quality^1^, ^2^, ^3^, ^4^, ^5^, ^6^, ^7^and expedite the planning process.^8^, ^9^, ^10^, ^11^ As a commercial KBRT optimization engine, RapidPlan (Varian Medical Systems, Palo Alto, CA) uses a pool of selected plans with consistent high quality as historical knowledge to train a DVH estimation model which predicts achievable DVH ranges and acceptable trade‐offs during the semi‐automatic plan optimization for the prospective patient.

Relative to the conventional experience‐based planning, superior or comparable results of RapidPlan have been reported in the preliminary applications to head and neck, lung, oesophageal, breast, hepatocellular, and prostate cancer patients.^12^, ^13^, ^14^, ^15^, ^16^, ^17^ However, consensus has been reached by these studies that both the model training and plan evaluation should be investigated further in a larger population and other cancer types in order to gain more experience and confidence before it is extensively applied clinically world‐wide.

This study retrospectively selected 150 preoperative simultaneous integrated boosting (SIB) VMAT plans that have been clinically approved and delivered for rectal cancer patients: 80 of them were manually refined and used to train the DVH estimation model, which was subsequently used to reoptimize the MLC sequences of the remaining 70 cases. Relative to the manually optimized clinical plans, dosimetric comparison was conducted to evaluate the performance of RapidPlan on semiautomated optimization of rectal VMAT plans with SIB.

## II. MATERIALS AND METHODS

### A. Plan selection

In accordance with the scope and clinical goals of this research, 150 manually optimized and consecutively treated plans were retrospectively selected. The gross target volume (GTV) was defined as the primary tumor, the mesorectal space, and the involved lymph nodes. The clinical target volume (CTV) was defined as the GTV, presacral region, mesorectal/lateral lymph nodes, internal iliac lymph node chain, and pelvic wall area.^(18)^ The CTV also covered the external iliac lymph nodes when anterior organ involvement was suspected, and covered the inguinal lymph nodes when the lower third of the vagina was invaded or major tumor extension into the internal and external anal sphincter was observed.^(19)^ The ${\text{PTV}}_{\text{boost}}$ and planning target volume (PTV) were created by adding an isotropic margin of 5 mm to the GTV and CTV, respectively. A total dose of 50.6 Gy and 41.8 Gy in 22 fractions was prescribed to 95% of ${\text{PTV}}_{\text{boost}}$ and PTV simultaneously.

Other planning goals included: a steep dose falloff from 50.6 Gy to 41.8 Gy in the external margin of 5 mm from ${\text{PTV}}_{\text{boost}}$ border into PTV (depending on the relative geometry of ${\text{PTV}}_{\text{boost}}$ and PTV); near maximum dose $D_{2\%}<107\%$ of 50.6 Gy (i.e., $D_{2\%}54.2\text{Gy}$); dose to 50% of femoral head $\left(D_{50\%\_\text{FH}}\right)$ and urinary bladder $\left(D_{50\%\_\text{UB}}\right)$ volumes <20.0 Gy and <30.0 Gy, respectively; and to minimize the mean dose to the femoral head ($D_{\text{mean}\_\text{FH}}$) and urinary bladder ($D_{\text{mean}\_\text{UB}}$). All plans were created using 10 MV photon, 1 full arc, ±10° collimator angle, and Millennium 120 MLCs based on Varian Trilogy accelerators.

### B. Model configuration and knowledge‐based treatment planning

Based on the Varian RapidPlan engine (V13.5), the anatomic structures, field geometries, dose matrices, and plan prescriptions of 80 aforeselected plans were extracted as historical knowledge to train a DVH‐estimation model.^(20)^ The ${\text{PTV}}_{\text{boost}}$ volumes ranged from 54.27 to 622.68 cm^3^ (mean±SD=179.46±93.60), and the PTV volumes ranged from 566.03 to 1688.26 cm^3^ (mean±SD=1209.25±181.82). Potential outliers as suggested by the statistical verification were examined and processed one by one, yet the diversity of OAR (organs at risk) geometries in the model were kept to accommodate the varieties of new patients.^16^, ^21^ The confirmed outliers were either removed, rematched, recontoured, or replanned by senior physicists to ensure only “good knowledge” was incorporated into the model and passed on to prospective plans.^(20)^

According to the manufacturer, the geometry‐based expected dose (GED) algorithm of RapidPlan divides the OARs into four subvolumes: the regions of out‐of‐field (scattered dose only), leaf‐transmission (MUs‐dependent), in‐field (modulated the most), and target overlap (comparable to the target dose) respectively.^(20)^ Therefore, the model is not intended for target dose estimations but works on the in‐field regions primarily; hence, the dose‐volume constrains for the targets were manually embedded to the model as fixed objectives, which were universally applied to all RapidPlan‐generated plans.

The remaining 70 plans were duplicated for testing the performance of the RapidPlan model. The ${\text{PTV}}_{\text{boost}}$ volumes ranged from 76.93 to 342.48 cm^3^ (mean±SD=177.23±75.02), and the PTV volumes ranged from 925.41 to 1941.6 cm^3^ (mean±SD=1243.24±199.82). Using the objectives generated by the model, the original MLC sequences were redesigned, while the other parameters such as the field geometry and photon energy were maintained. To evaluate the OAR exposure based on adequate and similar target dose coverage, both RapidPlan and the original plans were normalized to ensure 95% of both ${\text{PTV}}_{\text{boost}}$ and PTV were covered by their corresponding dose prescriptions. (Because normalization can be done on one target only in Eclipse, it was performed based on the more underdosed target; hence the other target may be slightly overdosed afterwards).

### C. Plan evaluation and statistical methods

The following metrics were evaluated to appraise the dosimetric difference between the knowledge‐based and experience‐based planning: 1) homogeneity index (HI) of ${\text{PTV}}_{\text{boost}}$ and PTV, defined as $\left(D_{2\%}–D_{98\%})/D_{50\%};2\right)$ conformity index of ${\text{PTV}}_{\text{boost}}$ $\left({\text{CI}}_{\text{PTVboost}}\right)$ and $\text{PTV}\;\left({\text{CI}}_{\text{PTV}}\right)$, defined as the volume enclosed by the corresponding prescription isodose surface divided by the target volume; 3) the relative volume of the hot spot exceeding 107% of prescribed dose in ${\text{PTV}}_{\text{boost}}(V_{107\%},i.e.V_{54.14\text{Gy}});4)$ the dose to the 50% of the femoral head and urinary bladder volume $(D_{50\%\_\text{FH}}$ and $D_{50\%\_\text{UB}});5)$ the mean dose to the femoral head and urinary bladder ($D_{\text{mean}\_\text{FH}}$ and $D_{\text{mean}\_\text{UB}}$); and 6) total monitor units (MU). Moreover, based on an in‐house MATLAB code (MathWorks, Natick, MA) and the DVH data exported in tabular format, the dose‐volume metrics were averaged over the 70 patients in each planning technique group for plotting comparison.

Based on SPSS (V 21.0), paired samples *t*‐test was used to compare the data couples when the normality test was passed, otherwise Wilcoxon signed‐rank test was performed to analyze the differences. The significance level was put to p < 0.05 (two‐tailed). All the plotting was performed by SigmaPlot software Version 10.0 (Systat Software, Inc., San Jose, CA).

## III. RESULTS

Table 1 displays the dosimetric statistics of the 80 cases for model training before (Training) and after replanning (Replanned) by the senior physicists during the model verification process. Much larger magnitude of dose reduction to the urinary bladder than to the femoral head was achieved by expert replanning.

Both RapidPlan and original plans were readily or nearly acceptable before the normalization. Only minor adjustment was performed for the coverage of ${\text{PTV}}_{\text{boost}}$ in 55 RapidPlan and 52 original cases, respectively. The rest of the plans were normalized for the coverage of PTV.

Table 2 lists the numerical statistics of the 70 patients as planned manually (original) or semiautomatically using model‐generated objectives (RapidPlan). The number of decimal places could not show the slight but significantly lower ${\text{HI}}_{\text{PTV}}$ of RapidPlan (0.255) than that of the original plans (0.263). As for the hot spot, positive $V_{107\%}$ was not observed in any RapidPlan cases, but appeared in 18 out of 70 original plans (25.70%): the greatest two $V_{107\%}$ values were 17.24% and 10.73%, respectively, and the rest were no larger than 2.28%. Limited by the subjective judgment, the suboptimal hot spots were deemed as acceptable trade‐offs at the time; which could have been avoided, however.

**Table 1:** Dosimetric statistics of the 80 training patients before (Training) and after replanning (Replanned) by senior physicists as a process of model verification. Dose unit (Gy)

				*95% Confidence Interval*		
		*Mean*	*SD*	*Lower*	*Upper*	p
${\text{HI}}_{\text{PTVboost}}$	Training	0.06	0.01	0.06	0.06	0.44
	Replanned	0.06	0.01	0.06	0.06	
${\text{HI}}_{\text{PTV}}$	Training	0.26	0.01	0.26	0.27	0.20
	Replanned	0.27	0.01	0.26	0.27	
${\text{CI}}_{\text{PTVboost}}$	Training	1.04	0.06	1.02	1.05	<0.01
	Replanned	1.08	0.07	1.06	1.09	
${\text{CI}}_{\text{PTV}}$	Training	1.04	0.03	1.03	1.05	<0.01
	Replanned	1.02	0.02	1.02	1.03	
$D_{50\%\_\text{FH}}$	Training	16.14	1.67	15.77	16.51	0.07
	Replanned	15.50	2.86	14.86	16.13	
$D_{\text{mean}\_\text{FH}}$	Training	17.38	1.65	17.02	17.75	0.03
	Replanned	16.83	2.10	16.37	17.30	
$D_{50\%\_\text{UB}}$	Training	28.19	3.41	27.44	28.94	<0.01
	Replanned	23.14	4.41	22.17	24.12	
$D_{\text{mean}\_\text{UB}}$	Training	29.15	2.73	28.55	29.75	<0.01
	Replanned	25.25	2.97	24.59	25.90	
$V_{107\%}$	Training	1.50	4.70	0.46	2.54	0.26
	Replanned	0.75	3.07	0.07	1.43	

HI= homogeneity index, CI= conformityindex, SD= standarddeviation, $D_{50\%}=$ dosetothe50%volumeofthestructure, $D_{\text{mean}}\;=$ meandose, FH= femoralhead, UB = urinarybladder, $V_{107\%}=$ volumereceivingover107%oftheprescribeddose.

**Table 2:** Dosimetric statistics of 70 patients as planned manually (original) or using RapidPlan‐generated objectives (RapidPlan). Dose unit (Gy)

				95 % Confidence Interval		
		*Mean*	*SD*	*Lower*	*Upper*	p
${\text{HI}}_{\text{PTVboost}}$	Original	0.06	0.01	0.06	0.06	<0.01
	RapidPlan	0.05	0.01	0.05	0.05	
${\text{HI}}_{\text{PTV}}$	Original	0.26	0.01	0.26	0.27	<0.01
	RapidPlan	0.26	0.26	0.25	0.01	
${\text{CI}}_{\text{PTVboost}}$	Original	1.00	0.05	0.99	1.01	0.05
	RapidPlan	0.04	0.99	0.98	1.00	
${\text{CI}}_{\text{PTV}}$	Original	1.03	0.02	1.02	1.03	0.90
	RapidPlan	1.03	1.02	0.02	1.03	
$D_{50\%\_\text{FH}}$	Original	15.52	2.17	15.00	16.03	<0.01
RapidPlan	13.99	1.16	13.71	14.26
$D_{\text{mean}\_\text{FH}}$	Original	16.59	2.07	16.10	17.08	<0.01
	RapidPlan	15.30	0.70	15.14	15.47	
$D_{50\%\_\text{UB}}$	Original	28.17	3.07	27.44	28.90	<0.01
	RapidPlan	23.24	2.13	22.74	23.75	
$D_{\text{mean}\_\text{UB}}$	Original	29.34	2.34	28.78	29.89	<0.01
	RapidPlan	25.40	1.36	25.08	25.73	
$V_{107\%}$	Original	0.46	2.42	0.00	1.04	<0.01
	RapidPlan	0.00	0.00	0.00	0.00	
MU	Original	402	39	392	411	<0.01
	RapidPlan	417	30	410	425	

HI = homogeneity index, CI= conformity index, SD = standard deviation, $D_{50\%}$ = dose to the 50% volume of the structure, $D_{\text{mean}}\;=$ mean dose, FH = femoral head, UB = urinary bladder, $V_{107\%}=$ volume receiving over 107% of the prescribed dose, MU=monitor units.

**Figure 1:**
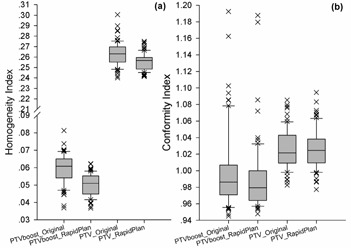
Distributions of the target dose homogeneity index and conformity index of the 70 testing patients, as planned using the conventional manual optimization (original) and model‐generated objectives (RapidPlan).

**Figure 2:**
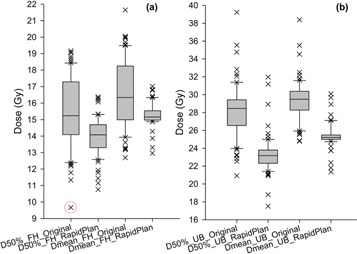
Distributions of $D_{50\%}$ and mean dose to the femoral head (FH) and urinary bladder (UB) of the 70 testing patients, as planned using the conventional manual optimization (original) and model‐generated objectives (RapidPlan).

The distributions of target HI and CI are displayed in Figs. 1(a) and 1(b), and the distributions of OAR dosimetry ($D_{50\%\_\text{FH}}$, $D_{\text{mean}\_\text{FH}}$, $D_{50\%\_\text{UB}}$, and $D_{\text{mean}\_\text{UB}}$) are displayed in Figs. 2(a) and 2(b), respectively. The bottom and top of the boxes, and the band in between, indicate the first and third quartiles, and the median value, respectively. The whiskers show the variability outside the upper and lower quartiles, the ends of which suggest the 10th and 90th percentiles, respectively. The outliers are plotted as individual crosses.

**Figure 3:**
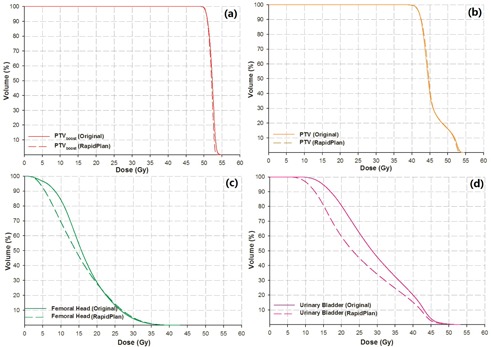
The mean DVHs of the 70 patients as planned using the conventional manual optimization (original, solid lines) and model‐generated objectives (RapidPlan, dashed lines).

Figure 3 shows the average DVHs of the 70 testing patients as planned by conventional method (solid lines) and knowledge‐based solution (dashed lines), respectively. Figs. 3(a), (b), (c), and (d) display the mean DVHs for ${\text{PTV}}_{\text{boost}}$, PTV, femoral head, and urinary bladder, respectively.

## IV. DISCUSSION

Consistent with previous published findings^8^, ^9^, ^10^, ^11^ the automatic process of RapidPlan has largely improved the planning efficiency; based on our stand‐alone workstation (two processors of 2.00 GHz, 32.0 GB RAM, 64‐bit Windows 7 Ultimate system), a typical case could be finished in about 30 min without any interactive objective adjustment.

For both the target (Fig. 1) and OAR (Fig. 2) metrics, the reduced degree of data dispersion in the RapidPlan group demonstrated its superior quality consistency and less variety than the original manual plans, which agreed with the RapidPlan rationale of alleviating the subjective dependency of individual planners.^1^, ^2^, ^3^, ^4^, ^5^, ^6^, ^7^

Improved OAR sparing by RapidPlan can be interpreted from the distributions of Fig. 2, except for a few outliers where the original plans achieved lower OAR exposure. As an example of explanation, the red‐circled data point of $D_{50\%\_\text{FH}}$ in Fig. 2(a) was optimized more aggressively in the original plan at a cost of higher dose to the urinary bladder, yet a balance was struck by the RapidPlan reoptimization. Of the 108 OAR outliers in the two groups (crosses in Fig. 2), 42 or 38.89% were from the same patients, indicating that these either “challenging” or “simple” cases for manual planning were also so for RapidPlan solution.

Quantitatively speaking (Table 2), minor but significant improvement (by 0.01) of dose homogeneity to ${\text{PTV}}_{\text{boost}}$ was observed in the RapidPlan group (p < 0.01), and the tiny difference (by 0.01) of ${\text{CI}}_{\text{PTVboost}}$ was insignificant (p = 0.05). No obvious disparity (by < 0.001) was found in ${\text{CI}}_{\text{PTV}}\left(p\;=\;0.90\right)$. These similar target dose metrics (also seen from the nearly overlapped target DVH lines in Figs. 3(a) and 3(b)) provided a fair basis for the comparison of OAR exposure, which is ascribed to the fact that RapidPlan does not estimate optimization objectives for the targets.

The overwhelmingly larger HI of PTV than that of ${\text{PTV}}_{\text{boost}}$ was attributable to the inherent features of SIB plans. However, the RapidPlan‐generated objectives have significantly reduced the HI_PTV_ by 0.008(p < 0.01), hence improved the dose gradient in this zone. A possible justification for this change was the inclusion of the transitional area (5 mm from ${\text{PTV}}_{\text{boost}}$ to PTV) as one of the “OARs” of our RapidPlan modeling rather than as a “target,” which predicts the DVH constraints (upper objectives) based on patient‐specific evaluations. The small magnitude of improvement might be ascribed to the huge geometric varieties of the transitional zone; the diverse volume, shape, and location of the targets may have largely complicated the model regression and estimation than was the case with the relatively regular OAR structures.

As shown in Figs. 3(c) and 3(d) and Table 2, RapidPlan has significantly (p < 0.01) reduced the dose to the femoral head by 1.53 Gy (9.86%) and by 1.29 Gy (7.78%) for the $D_{50\%}$ and $D_{\text{mean}}$, respectively. For the urinary bladder, the reduction magnitudes for the $D_{50\%}$ and $D_{\text{mean}}$ were 4.93 Gy (17.50%) and 3.94 Gy (13.43%), respectively $D_{\text{mean}}$. RapidPlan also showed a stronger capability of controlling the hot spots ($V_{107\%}$). These improvements were achieved at a cost of slightly but significantly more MUs consumed by the RapidPlan cases (increased by 15 MU or 3.73%, p < 0.01). The apparently “unavoidable” trade‐offs or “hard‐to‐achieve” goals of OAR sparing during the manual optimization were well managed by RapidPlan automatically, suggesting its potential value for overcoming the subjective limitations that arise during the conventional trial‐and‐error iteration of manual planning. These observations were highly consistent with earlier prostate studies at a similar clinical site. RapidPlan‐associated dose reduction to the bladder^7^, ^13^ and femoral head^7^, ^12^, ^13^ were macroscopic and significant. Similar improvement of OAR sparing and consistency has also been widely reported in other clinical sites.^14^, ^15^, ^16^, ^17^

Should the performance of RapidPlan be strongly dependent on the expertise of the original planner and the model developer, a greater model library consisting better plans may further enhance the results of knowledge‐based planning.^(22)^ Replanning by senior physicists significantly improved the quality of training plans during the model verification process (Table 1); that, however, is sorely time‐consuming and clinically impractical for all prospective patients. By means of machine learning, RapidPlan successfully applies the expertise of senior physicists onto prospective patients to generate above‐average plan quality without interactive human intervention. Relative to the clinical plans, an even larger magnitude of dose reduction to the femoral head was achieved by RapidPlan than by the best effort of manual replanning (Tables 1 and 2).

“Varian‐provided models,” as preconfigured by the world‐leading institutes, should be of high quality; however, modifications accommodating custom contouring and planning protocols were not allowed for these modules. This nonrevisability also prevents new representative cases from being added to the library in order to enlarge the scope of model applications. As other potential limitations of RapidPlan, some critical parameters such as the beam energy and field geometry are not optimizable by the model, which may vary dramatically among different centers and planners. That was why custom models were more popular in the previous publications.^12^, ^13^, ^14^, ^15^, ^16^, ^17^ As for this study, the historical plans represent the average quality that is currently and reasonably achievable, and the improvement may be mostly attributable to the personalized objective as estimated by RapidPlan, which passes on the expertise as incorporated in the model to prospective plans.

## V. CONCLUSIONS

Using a DVH estimation model trained with 80 historical VMAT SIB plans of high quality for rectal cancer patients, the knowledge‐based replanning of 70 clinical plans displayed significantly superior characteristics compared to those of the conventional manual planning in terms of target dose falloff, hot spot control, and OAR sparing. Suboptimal manual plans could be improved by the RapidPlan model, hence enhancing the consistency of plan quality.

## ACKNOWLEDGMENTS

This work was supported by National Natural Science Foundation of China (11505012, 81402535, 81472814), Beijing Municipal Administration of Hospitals’ Youth Programme (code: QML20151004), and Special Fund for Quality Scientific Research in the Public Welfare (201510001–02). The authors thank Jian Gong, Zhongsu Feng, Qiaoqiao Hu, Zhuolun Liu, and Jian Zhang for their assistance.

## COPYRIGHT

This work is licensed under a Creative Commons Attribution 3.0 Unported License.

## Supporting information

Supplementary Material
