# Supplementary material for: A dosimetric evaluation of knowledge‐based VMAT planning with simultaneous integrated boosting for rectal cancer patients
Source: J Appl Clin Med Phys. 2016 Nov 8;17(6):78–85. doi: 10.1120/jacmp.v17i6.6410 (PMC5690500; doi:10.1120/jacmp.v17i6.6410)
Supplement: Supplementary file 1 — Supplementary Material [file ACM2-17-078-s001.docx]

Title Page

**A dosimetric evaluation of knowledge-based VMAT planning with simultaneous-integrated-boosting for rectal cancer patients**

**Hao Wu^*^, Fan Jiang^*^, Haizhen Yue, Sha Li, Yibao Zhang ^a^**

*Key laboratory of Carcinogenesis and Translational Research (Ministry of Education/Beijing), Department of Radiation Oncology, Peking University Cancer Hospital & Institute, Beijing, 100142. China*

*ybzhang77@gmail.com*

* Hao Wu and Fan Jiang contributed equally to this work.

^a^ Corresponding author: Yibao Zhang, Department of Radiation Oncology, Beijing Cancer Hospital, 52 Fucheng Road, Haidian, Beijing 100142 China; Phone: +8610-88196033; Fax: +8610-88196033; Email: ybzhang77@gmail.com

Running title: Knowledge-based rectal SIB VMAT
